# Follow-up after the Ross procedure, how significant it is, case reports of three patients

**DOI:** 10.1186/s13019-015-0369-8

**Published:** 2015-11-03

**Authors:** Panagiotis Artemiou, Ingrid Schusterova, Alzbeta Tohatyova, Jozefina Cocherova, Peter Krcho, Frantisek Sabol

**Affiliations:** 1 Department of Cardiac Surgery, East Slovak Institute for Cardiovascular Diseases, Medical Faculty of the University of Pavol Jozef Safarik, Kosice, Slovakia; 2 Department of Paediatrics and Adolescent Medicine, Medical Faculty of the University of Pavol Jozef Safarik, Kosice, Slovakia; 3 Department of Neonatology, Pavol Jozef Safarik University, Kosice, Slovakia

**Keywords:** Ross procedure, Follow-up

## Abstract

**Background:**

Dilatation of the pulmonary autograft is a major drawback of the Ross procedure and it is the leading cause for reoperation in these patients.

**Case Presentation:**

In this report we describe 3 cases reports, each one with a different outcome, of patients that underwent the Ross procedure.

**Conclusions:**

In order to prevent any lethal or non-lethal complications of the pulmonary autograft these patients need a close and life- long systematic follow-up.

## Background

Replacement of the aortic valve or aortic root with a pulmonary autograft (Ross procedure) and replacement of the pulmonary valve with a pulmonary allograft or xenograft was first described in 1967 by Ross [1] and is now a widely used technique for the treatment of aortic valve disease in the children and young adult. It provides a viable valve with potential advantages, including excellent hemodynamic function, ability to grow and durability with no need for anticoagulation. Excellent short and midterm results have been demonstrated with low operative mortality and low rates of valve-related deaths and complications [1, 2].

Although the pulmonary valve is known to adapt to the new environment there is continuing concern about the risk of long-term autograft dilation and aortic regurgitation which occurs in 10–30 % of late survivors [2].

In order to prevent any potential complications from the autograft dilation and the aortic regurgitation these patients need a close and systematic follow-up.

In this study we briefly describe three cases reports from patients from our database after the Ross procedure with a different outcome in each report, in order to raise the significance of the follow-up in this group of patients.

The registry of our institute has eight patients, five female and three male patients. The study period was from 2000 until today. The age of the patients ranges from 18 to 37 years old. One patient was lost from the follow-up and from the remaining, six patients are alive and one male patient has died from sudden cardiac death due to aortic aneurysm rupture. Two patients, one female and one male patient underwent redo operations. The female patient underwent a redo-Bentall operation 12 years after the Ross procedure due to aortic dilatation and severe aortic regurgitation and the male patient underwent aortic and pulmonary valve replacement 15 years after the Ross procedure due to severe aortic and pulmonary valve regurgitation.

All the patients in the childhood underwent the Ross procedure due to congenital aortic valve stenosis/regurgitation and all the operations were performed in a single center. In all cases the Ross operation was performed as an autograft root replacement without any reinforcement procedures. Standard techniques of cardiopulmonary bypass were used, with bicaval cannulation, moderate hypothermia and antegrade and retrograde cold potassium cardioplegia. All patients had intra-operative trans-esophageal echocardiography.

During the follow-up the patients had a physical examination approximately every 6 months and echocardiography once a year. The examinations were done by the local cardiologists with no experience with such patients, and sometimes not by the same cardiologist. The echocardiographic examinations were done according the guidelines of the American Society of Echocardiography [3]. Cardiac CT (computer tomography) examination was done in the cases that the patient was referred to a redo surgery and due to limitations of the transthoracic echocardiography in one patient. The role of the preoperative CT was to determine the aortic diameter and to show the relationship between the sternum and the right ventricle.

During the follow-up all the patients had a progressive dilation of the aorta with the diameter in the latest follow-up to range from 34 mm to 56 mm with also a progressive aortic regurgitation. Because of this, two patients were referred to a redo surgery and one patient had a sudden cardiac death. Moreover one patient developed severe pulmonary valve regurgitation, four patients developed mild to moderate pulmonary valve stenosis and one patient developed severe pulmonary stenosis. All the surviving patients are on NYHA class I–II.

Concerning the medical treatment, all these were young patients with no hypertensive disease and they could not tolerate any beta blocker treatment. Only in cases of hyperdynamic circulation with sinus tachycardia they were administered.

Below we describe three representative cases of these patients.

## Case presentation

### Case report 1

A 24 year old female patient in 2002 at the age of 12 underwent a Ross procedure due to severe congenital aortic valve stenosis. During the follow-up, in the last year the aortic root dilated from 30 mm to 34 mm. Other findings were mild aortic regurgitation and mild pulmonary valve stenosis (mean gradient 15 mmHg) with no progression. The patient is still under surveillance and clinically is on NYHA class II.

### Case report 2

**Fig. 1:**
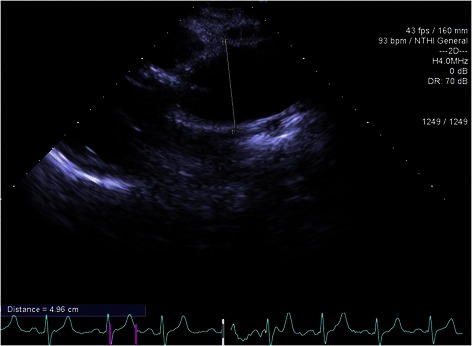
Case report 2, transthoracic echocardiogram parasternal long axis- ascending aorta before the operation

A 29 year old female patient in 2002 at the age of 17 underwent a Ross procedure due to a congenital severe aortic valve stenosis combined with regurgitation. During the follow-up the aortic root and the ascending aorta gradually dilated to 52 mm and 50 mm respectively with also mild to moderate aortic regurgitation (Fig. 1).

In 2014 she underwent a redo-Bentall operation with the replacement of the dilated aorta and aortic valve with a mechanical conduit ATS 22 mm/26 mm (Medtronic Inc MN USA). After the operation the patient is stable and is doing well.

### Case report 3

A male patient in the year 2000 at the age of 10 underwent a Ross procedure due to severe aortic valve regurgitation combined with stenosis. During the operation also annuloplasty of the aortic ring was done because of the difference between the native aortic ring and the pulmonary autograft diameters. In 2007 he underwent balloon dilation of the pulmonary valve due to severe stenosis with a decrease of the peak gradient from 80 mmHg to 30 mmHg. During the follow-up aortic root and ascending aorta gradually dilated to 50 mm and 56 mm respectively. Aortic regurgitation was mild. Moreover the pulmonary stenosis after the balloon dilatation also deteriorated with a mean gradient of 57 mmHg. The patient suffered from pulmonary hypertension (PAP 52 mmHg) and moderately dilated right ventricle. In 2010 the patient was referred to surgery for replacement of the aortic root, the ascending aorta and the pulmonary valve. Unfortunately, the patient died from aortic rupture before surgery during the preoperative evaluation. The event took place in the hospital and the patient was rushed to the operating room where the cause of death was found.

## Discussion

Pulmonary autograft dilatation is common after the Ross procedure in late survivors. The dilation progresses over time and is often accompanied by dilatation of the native aorta [2]. Dilatation of the neosinuses of Valsalva occurs in 10–30 % of late survivors, especially when a freestanding autograft root replacement is used [2]. A recent report by Ruzmetov M et al. [1] showed that dilatation of the pulmonary autograft was the most common indication for autograft reoperation with a median interval of 8 years after the original operation. Moreover autograft dilatation was also the leading cause of reoperation in a series of Juthier F et al. [2]. The interval between the initial procedure and the reoperation was 7.2+/− 5 years.

It is obvious that the patients after the Ross procedure need a close life-long systematic follow-up, in order to prevent any complication from the pulmonary autograft dilation. The follow-up of these patients should be done in a specialized center by the same medical personnel each time, experienced with this group of patients. For example the serial echocardiographic or computer tomography measurements of the neoaorta dimensions should be done by the same cardiologist or radiologist respectively in order to minimize any errors such as the inter-reader variability between the measurements.

In contrast, the patients from our registry had the follow-up examinations done at the local hospital and in some cases by different medical personnel. In our opinion, this may lead to delayed diagnosis of any changes of the neoaorta dimensions and delayed reference to prophylactic surgery. To support this, the third case report described a patient who died from aortic rupture while waiting for operation.

All the patients after the Ross procedure should be evident in a central database. In case of the patients non-compliance with the scheduled follow-up visit, the patient should be traced and invited to undergo the examination.

The preferred imaging modality used for the follow-up in our center was the transesophageal echo, and the follow-up was done approximately on a yearly basis. Cardiac CT was done in the cases that the patients were referred to a redo surgery.

The 2010 AHA/ACC guidelines for the diagnosis and management of patients with thoracic aortic disease [4] suggested that the follow-up interval after aortic root and ascending aorta procedures should be a baseline transthoracic echocardiographic examination before discharge and then on yearly basis.

The 2014 ESC guidelines for the diagnosis and treatment of aortic diseases [5] suggested that for patients undergoing surgical thoracic aortic repair the first follow-up should be performed 1 month after the treatment and surveillance should be repeated after 6 months, 12 months, and then yearly. Computer tomography (CT) is the modality of choice, but magnetic resonance imaging (MRI) or transthoracic/transesophageal echocardiography (TTE/TOE) may be used as alternative modalities.

Finally Evangelista A [6] suggested after surgery imaging control by TTE/TOE should be performed before discharge and a TTE annually from then on. In cases with an ascending aorta diameter > 45 mm MRI or CT should be performed every 2–3 years.

In our group of patients we could not isolate any factors which may justify the diverse outcome in the different patients. It is a small registry and we can not have definite conclusions. Moreover all the operations were performed with the same surgical technique, all the patients had the same demographic characteristics and the treatment of a beta-blocker in some patients with a hyperdynamic circulation did not have any influence on the aneurysm growth rate. Recently in a review, the role of the beta-blocker in the prevention of aortic aneurysms was questioned [7].

Finally, surgical stabilization and reinforcement techniques were reported to preserve autograft function and resulted in significantly lower reoperation rates [8].

## Conclusion

In conclusion, dilatation of the pulmonary autograft is a major drawback of the Ross procedure and it is the leading cause for reoperation. In order to prevent any lethal or non-lethal complications of the pulmonary autograft these patients need a close and life-long systematic follow-up.

## Consent

The consents of the three patients was obtained for presentation of these reports.

## References

[1] Ruznetov M, Welke KF, Geiss DM, Buckley K, Fortuna RS (2014). Failed autograft after the ross procedure in children: management and outcome. Ann Thorac Surg.

[2] Juthier F, Vincentelli A, Pincon C, Banfi C, Ennezat PV, Marchaux S (2012). Reoperation after the ross procedure: incidence, management and survival. Ann Thorac Surg.

[3] Lopez S, Colan SD, Frommelt PC, Ensing GJ, Kendall K, Younoszai AK (2010). Recommendations for quantification methods during the performance of a pediatric echocardiogram. A report from the pediatric measurements writing group of the American Society of Echocardiography. Pediatric and congenital heart disease group. J Am Soc Echocardiogr.

[4] Hiratzka LF, Bakris GL, Beckman JA, Bersin RM, Carr VF, Casey DE (2010). ACCF/AHA/AATS/ACR/ASA/SCA/SCAI/SIR/STS/SVM guidelines for the diagnosis and management of patients with thoracic aortic disease: executive summary. Circulation.

[5] Erbel R, Adoyans V, Boileau C, Bossone E, Di Bartolomeo R, Eggebrecht H (2014). ESC guidelines on the diagnosis and treatment of aortic diseases. Eur Heart J.

[6] Evangelista A (2014). Imaging aortic aneurismal disease. Heart.

[7] Chun AS, Elefteriades JA, Mukherjee SK (2013). Do β-blockers really work for prevention of aortic aneurysms?. Aorta.

[8] Charitos EI, Hanke T, Stierle U, Robinson DR, Bogers AJJC, Hemmer W (2009). Autograft reinforcement to preservr autograft function after the Ross procedure. A report from the German-Dutch registry. Circulation.

